# Vitamin D and Its Role During Pregnancy in Attaining Optimal Health of Mother and Fetus

**DOI:** 10.3390/nu4030208

**Published:** 2012-03-21

**Authors:** Carol L. Wagner, Sarah N. Taylor, Adekunle Dawodu, Donna D. Johnson, Bruce W. Hollis

**Affiliations:** 1 Division of Neonatology, Department of Pediatrics, Medical University of South Carolina, 173 Ashley Avenue, MSC 513, Charleston, SC 29425, USA; Email: taylorse@musc.edu (S.N.T.); hollisb@musc.edu (B.W.H.); 2 Global Health Center, Cincinnati Children’s Hospital Medical Center, Cincinnati, OH 45229, USA; Email: Adekunle.Dawodu@cchmc.org; 3 Division of Maternal-Fetal Medicine, Department of Obstetrics and Gynecology, Medical University of South Carolina, Charleston, SC 29425, USA; Email: johnsodo@musc.edu

**Keywords:** vitamin D, cholecalciferol, calcitriol, pregnancy, neonate

## Abstract

Despite its discovery a hundred years ago, vitamin D has emerged as one of the most controversial nutrients and prohormones of the 21st century. Its role in calcium metabolism and bone health is undisputed but its role in immune function and long-term health is debated. There are clear indicators from *in vitro* and animal *in vivo* studies that point to vitamin D’s indisputable role in both innate and adaptive immunity; however, the translation of these findings to clinical practice, including the care of the pregnant woman, has not occurred. Until recently, there has been a paucity of data from randomized controlled trials to establish clear cut beneficial effects of vitamin D supplementation during pregnancy. An overview of vitamin metabolism, states of deficiency, and the results of recent clinical trials conducted in the U.S. are presented with an emphasis on what is known and what questions remain to be answered.

## 1. Introduction

In pregnancy, to understand the place and importance of micronutrients or any nutrient, we must go back to the beginning—at the time when the sperm fertilizes the egg and implantation 5–6 days later into the uterine wall. Seemingly on “autopilot”, the maternal milieu may affect the well-being of not only that developing embryo and later fetus, but generations of offspring to come. The circumstances of various famines, plights, and poverty during recent human existence show us this [1,2,3]. Yet, it requires painstaking and meticulous research to understand the role that maternal nutrition during conception and later pregnancy plays on fetal well-being. 

Much of what we know comes from observation and extrapolation from clinical patients with known nutrient imbalance or metabolic dyscrasias, from those afflicted with famine, and from animal studies. Yet, in those instances, we tend to focus on one nutrient and its effects if lacking or in excess. It is elementary, however, to think that one nutrient or micronutrient does not interact with other nutrients and chemicals within the body; instead of acting in isolation, there is a synergistic effect. Within the framework of the living being, there is a developmental and aging cascade that is not static, but which is in flux, such that findings on a given day may be valid for that day but become less applicable with additional growth or with the upregulation of involved enzymatic systems. While the complexity of what we are trying to predict and to understand may be evident with closer examination, we tend to generalize and to think that we know all we could about a given nutrient or micronutrient. Yet, decade after decade of scientific inquiry shows us not so much how right we are but how little we know in the grand scheme. It is with these cautions and confessions that we approach this review about vitamin D’s role during pregnancy.

In the sections ahead, we will focus on one micronutrient—vitamin D—and will discuss what we know and what we surely do not know in the context of the pregnant woman and her developing fetus. During this foray into the world of vitamin D and the fetus, we must first define what vitamin D is, what it is not, and review the metabolic pathways linked with vitamin D.

## 2. What Is Vitamin D?

Vitamin D is a preprohormone that is made by most living plants and terrestrial animals. In the true sense of the word, vitamin D is not a “vitamin” because the main source of vitamin D is that which we synthesize ourselves—in our skin—with less than 10% coming from dietary sources. Vitamin D comes in two major forms—vitamin D_2_ or ergocalciferol and vitamin D_3_ or cholecalciferol. While certain plants are capable of making both forms of vitamin D, the major form made by plants is vitamin D_2_ following ultraviolet B exposure of the provitamin D_2_ ergosterol [4,5]. In comparison, humans can metabolize both vitamin D_2_ and D_3_, but can only synthesize *de novo* vitamin D_3_. (For the purposes of this review, unless otherwise mentioned, when vitamin D and its metabolites are stated without a subscript, both families of vitamin D—cholecalciferols and ergocalciferols are described.)

## 3. Sources of Vitamin D

**Figure 1:**
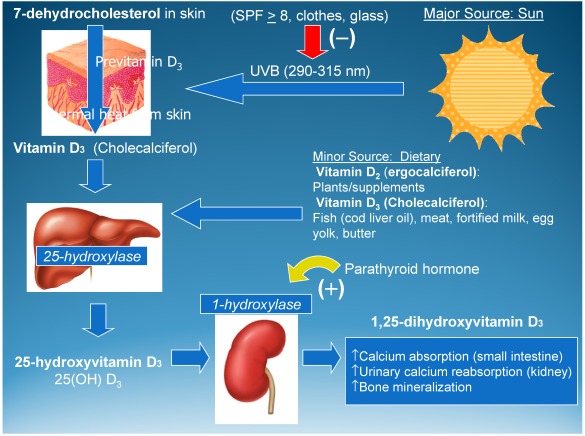
Human vitamin D synthesis pathways. Reproduced with permission from [7].

As shown in Figure 1, the *de novo* synthesis of vitamin D_3_ in humans and other animals begins in the skin with the parent compound 7-dehydrocholesterol or provitamin D_3_. Following exposure to ultraviolet B radiation in the range of 280–320 nm, 7-dehydrocholesterol becomes previtamin D_3_. Through a subsequent thermal reaction in the skin, previtamin D_3_ is isomerized into vitamin D_3_. It is important to note that unlike other steroid hormones in the body whose main substrate is cholesterol, vitamin D synthesis requires the 7-dehydrocholesterol precursor and sunlight at a specific wavelength and angle. Without this reaction, humans are dependent on dietary intake of vitamin D, which may be in the form of either vitamin D_2_ or D_3_. A western diet, however, provides less than 200 IU (5 μg) vitamin D/day, a point that was reestablished in our two recently completed vitamin D clinical trial during pregnancy [6]. 

## 4. General Vitamin D Metabolism

In order to understand the differences between the nonpregnant and pregnant states and their effects on vitamin D metabolism, it is essential to understand the nonpregnant state first. Following its synthesis, vitamin D binds to vitamin D binding protein (VDBP) and finds its ways into the circulation. Dietary and endogenous vitamin D appear to act similarly with half-life between 12 and 24 h, the length of time depending on how quickly the liver converts vitamin D to 25-hydroxy-vitamin D (also known as calcidiol). Vitamin D is measured in international units (IU) or micrograms with a known conversion of 40 IU equal to 1 microgram. 

While there appears to be a differential conversion rate of the two forms of vitamin D to 25(OH)D [8], the conversion of either form is dependent on a functional liver and the activity of 25-hydroxylase. Thus, those with impaired liver function will have diminished conversion of vitamin D to 25(OH)D. Following its synthesis, 25(OH) then enters the circulation where it is tightly bound to VDBP. Only a small amount of 25(OH)D is unbound or “free”. The half-life of 25(OH)D is 2–3 weeks, making it a much better indicator of the body’s vitamin D status than vitamin D. Of note, 25(OH)D can be expressed as ng/mL or nmol/L. The conversion from ng/mL to nmol/L is 2.5; thus, a 25(OH)D concentration of 20 ng/mL equals 50 nmol/L.

Once 25(OH)D is formed in the liver, it enters the circulation. Best known is the processing of 25(OH)D by the kidney where 25(OH)D complexed with VDBP and megalin is taken up by the epithelial cells of the proximal tubules and converted to the active hormonal form of vitamin D—di-hydroxy-vitamin D (1,25(OH)_2_D or calcitriol)—by the action of the mitochondrial enzyme 1-α-hydroxylase. 

1,25(OH)_2_D’s endocrine effects include the following classic triad of action: (1) increase intestinal calcium (as Ca^2+^ ions) absorption through the actions of calbindin; (2) increase urinary calcium reabsorption; and (3) regulation of parathyroid hormone in a negative feedback loop that allows calcium to be absorbed from the gastrointestinal tract, reabsorbed from urine, and metabolized from bone in order to maintain calcium homeostasis within the body. Because calcium is essential to all tissues and organs, particularly the heart, skeletal muscle and brain, the body will claim calcium if necessary from the skeleton. Adequate vitamin D must be on hand to provide enough substrate to form 25(OH)D, which in turn, is converted to 1,25(OH)_2_D, whose half-life is 8 hours. In individuals with vitamin D deficiency, only trace amounts of vitamin D will be found in the body because whatever comes into the circulation is quickly converted to 25(OH)D and then to 1,25(OH)_2_D to maintain calcium homeostasis.

## 5. Extrarenal Effects of Vitamin D: Modulating Immune Function

For decades, it was thought that only the kidney has the capacity to metabolize 25(OH)D; however, extrarenal metabolism has been demonstrated in every organ system in the body [9,10,11,12]. During pregnancy, the placenta is probably the most prominent site for extra-renal activation of vitamin D [13]. It appears that the extrarenal function of vitamin D has more to do with immune function than with calcium metabolism and homeostasis. Support for this premise first came from the observations of Mellanby and others at the turn of the 20th century: during that time, Mellanby in his study of rachitic children and dogs noted an increased risk of respiratory infections in those afflicted [14,15]. Additional reports came from those working with tuberculosis patients and the beneficial effect of being in sunlight and outdoors in the treatment of the condition [16]. Weick [17] in 1967 and Rehman [18] in 1994 independently observed that children with rickets appeared ill, with decreased energy and activity, and were more susceptible to respiratory illnesses. Despite these observations, it was concluded that the condition of vitamin D deficiency led to weakness and malnutrition and was not a direct effect of vitamin D on the immune system. The mechanism of action of these processes and health derangements would not be understood until the advent of molecular biology.

Vitamin D appears to affect immune function in two ways: (1) upregulation of the innate immune system; and (2) downregulation of the adaptive immune system. Focusing on the innate immune system first, a major mechanism of action of vitamin D is via an endogenous antimicrobial peptide called cathelicidin (LL-37), which is generated in response to microbial invasion through activation of toll-2 receptors (TLR) on monocytes and macrophages [19,20,21]. Not surprisingly, the vitamin D receptor element (VDRE) is contained in the promoter region of the gene for LL-37. VDRE are found only in the LL-37 gene promoters of primates, suggesting that the ability of vitamin D to promote LL-37 antibacterial action is a relatively recent event in evolution. Both 1,25(OH)_2_D and 25(OH)D have the ability to induce the expression of cathelicidin in monocyte/macrophage and epidermal lineage in cells that simultaneously have the 25(OH)D hydroxylase [22]. 

Significant support for the role of vitamin D in immune processes and function came in 2006 when Liu *et al.* published their landmark study in *Science* [19]. Serum samples taken from African American subjects with low 25(OH)D were inefficient in supporting cathelicidin mRNA induction; however, with the addition of 25(OH)D to those samples with low 25(OH)D levels this pattern was reversed. Thus, in this series of experiments, the addition of 25(OH)D_3_ restored the ability of sera from individuals with low 25(OH)D concentrations to support TLR2/1L-mediated induction of cathelicidin mRNA. A related study by Fabri *et al.* [23], showed that IFN-γ-mediated antimicrobial activity of human macrophages, especially important in HIV and tuberculosis patients, is dependent on vitamin D. Both study findings have implications for the pregnant woman and her developing fetus, but our understanding of such processes following maternal exposure to a pathogen or maternal infection remains scant. There is every reason to suggest that such processes are fully functional in the pregnant woman. 

Vitamin D’s role as a modulator of the immune system encompasses the adaptive immune system as well. 1,25(OH)_2_D not only has the ability to affect processes within macrophages and monocytes, but also in T and B lymphocytes as well. The vitamin D receptor (VDR) is found on activated (but not resting) human T- and B-lymphocytes. Whereas 1,25(OH)_2_D appears to activate the bacteriocidal process within macrophages and monocytes, it has different effects, that include suppression of T-cell proliferation and modulation of T-cell phenotype—with anti-inflammatory properties [24]. By binding to the VDR on T cells, 1,25(OH)_2_D acts to: (1) inhibit the proliferation of uncommitted T_H_ (helper) cells and (2) promote the proliferation of immunosuppressive regulatory T cells, or T_reg_S, with notable accumulation of these cells at sites of inflammation [22]. It appears that 1,25(OH)_2_D suppresses certain B cell functions such as proliferation and immunoglobulin production and retards the differentiation of B-lymphocyte precursors to mature plasma cells *in vitro*. These *in vitro* findings help to explain the significant association between vitamin D deficiency and autoimmune diseases, such as systemic lupus erythematosus [25], multiple sclerosis [26,27,28,29,30,31,32,33,34,35], rheumatoid arthritis [31,36,37], diabetes—both types 1 [31,38,39,40,41,42] and 2 [43,44,45], and certain cancers, such as colon [46,47,48,49], breast [50,51,52,53,54,55], and prostate [55,56,57,58,59]. Additionally, the role of vitamin D in immune function intensifies the need to establish vitamin D sufficiency during pregnancy. 

## 6. Role of Parathyroid Hormone During Pregnancy

Throughout the lifecycle, parathyroid hormone or PTH’s main function is to maintain calcium homeostasis: PTH levels are inversely related to calcium concentrations in the blood, increasing when there is a decrease, and decreasing when there is an increase in calcium, acting through the parathyroid hormone 1 receptor (high levels in bone and kidney) and the parathyroid hormone 2 receptor (high levels in brain, placenta, and other endocrine tissues). In nonpregnant adolescents and adults, increased PTH is associated with lower circulating concentrations of ionized calcium, which is most often precipitated by vitamin D deficiency and decreased intestinal calcium absorption. Thus, PTH levels are related to vitamin D status as well: PTH upregulates 1-α-hydroxylase, the enzyme responsible for 1-α-hydroxylation of 25(OH)D, converting 25(OH)D to 1,25(OH)_2_D. In individuals who have vitamin D deficiency, the body increases production of PTH to maintain 1,25(OH)_2_D and calcium homeostasis. If vitamin D deficiency is sustained, secondary hyperparathyroidism will result. 

It is interesting that during pregnancy alone and at no other time during the lifecycle there is an uncoupling of vitamin D metabolism from calcium such that by the end of the first trimester, 1,25(OH)_2_D levels are more than double what they are during the nonpregnant state without concurrent changes in serum calcium concentrations [6]. It is not surprising, then that during pregnancy PTH as a marker of vitamin D status is a less reliable predictor than in nonpregnant adults [60,61,62]. Haddow et al, in their study of 430 African American and 586 Caucasian pregnant women living in Rhode Island at latitude 42° N, found a weak negative correlation between total circulating 25(OH)D and PTH (*r* = −0.074 and −0.137 for Caucasian and African American women, respectively), affected somewhat by seasonality. 

An earlier study by Okonofua *et al.* [63], in their study of healthy Asian and Caucasian pregnant women showed an inverse correlation between maternal serum 25(OH)D and PTH (*r* = −0.32, *p* < 0.002), a relationship that did not persist when the Asian and Caucasian women were considered separately. Not unexpectedly, there was an inverse relationship between calcium and PTH in the same women (*r* = −0.51, *p* < 0.0001); suggesting that serum calcium alone has a more profound effect on PTH during pregnancy. In contrast, Morley *et al.* [64] and Hamilton *et al.* [65] both reported slightly lower correlations between PTH and 25(OH)D (*r* = −0.18 (*p* < 0.001) and *r* = −0.24 (*p* < 0.001)), respectively, than did Okonofua *et al.* [63], but higher correlations than Haddow *et al.* [60]. While Okonofua, et al, suggest that normalization of maternal calcium and PTH would be useful endpoints in determining the success of vitamin D supplementation during pregnancy, more recent studies suggest that because PTH threshold estimates cannot be precisely defined, total circulating 25(OH)D alone is a better measure of maternal vitamin D status [60,64,65].

In our recently published National Institute of Child Health and Human Development (NICHD)-sponsored trial involving 350 women randomized to one of three treatment groups (400 (10 μg), 2000 (50 μg) or 4000 (100 μg) IU vitamin D_3_/day) who were followed from 12 weeks of gestation until delivery, there was a trend in all subjects of PTH being higher as the subjects progressed through pregnancy, but was not significantly different by treatment [6]. Decreases in circulating PTH were observed if the levels attained were analyzed by race only. The African American group clearly had decreased circulating PTH as circulating 25(OH)D levels increased. As with Haddow *et al.* [60], threshold estimates were not precisely defined for the three racial/ethnic groups or by treatment group. 

## 7. Vitamin D Deficiency

Vitamin D deficiency is best understood in terms of bone disease: rickets during infancy and early childhood and osteopenia and osteoporosis in adulthood. With rickets, bone formation and development is seriously altered with severe vitamin D (*i.e.*, substrate) deficiency. Beyond childhood, severe vitamin D deficiency can occur in young women, including those who are pregnant, with higher risk with advancing age in a woman’s lifecycle. While there can be some calcium loss during pregnancy through fetal demands and increased urinary calcium excretion which increases with advancing pregnancy, there is a rebound effect such that multiparous women are not at increased risk of osteopenia compared with nulliparous women. Throughout gestation, if a woman is vitamin D deficient, it appears to impact fetal bone health more than maternal [66,67,68]. In addition, based on rickets and osteoporosis data, it appears that bone health, which is linked with renal production of 1,25(OH)_2_D and calcium metabolism is maintained at significantly lower levels of circulating vitamin D concentrations compared to other health factors such as immune function, which is dependent on delivery of 25(OH)D to cells of the target tissue. Thus, there are different deficiency set points: the risk of rickets increases significantly when total circulating 25(OH)D falls below 10 ng/mL (25 nmol/L) whereas cathelicidin mRNA expression as a marker of immune function continues to be suppressed until 25(OH)D circulating levels reach at least 20 ng/mL (50 nmol/L) [69].

What was striking in the previously mentioned independent studies by Haddow *et al.* [60], Hamilton *et al.* [65], and the NICHD vitamin D supplementation trial [70,71] was the high incidence of vitamin D deficiency during early pregnancy in women with darker pigmentation. Using the recently revised Institute of Medicine’s (IOM) 2010 criterion for vitamin D deficiency of total circulating 25(OH)D < 20 ng/mL (50 nmol/L) [72], approximately 70% of the African American compared to 35% of Caucasian women in the Rhode Island study had evidence of vitamin D deficiency during winter, with slight improvement during summer months compared to winter months in both groups. In the study by Hamilton *et al.* [65], 68% African American and 33% Hispanic women living at latitude 32°N were deficient compared to 18% Caucasian women. Similarly, in the NICHD trial conducted in Charleston, SC, 75% African American, 30% Hispanic and 12% Caucasian women met the definition of vitamin D deficiency set forth by the IOM [70]. Yet, as mentioned earlier, based on the NICHD trial results, the optimization of 1,25(OH)_2_D production does not occur until total circulating 25(OH)D levels are at least 40 ng/mL (100nmol/L) [6]. Applying this new criterion to the data suggests that, with the present Western diet and lifestyle, without adequate supplementation, virtually all unsupplemented African American and many Hispanic women in the United States do not have optimal 1,25(OH)_2_D production. The significance of this is highlighted in the following sections of this review. 

## 8. A Global Perspective on Vitamin D Status of Women During Pregnancy

With improved laboratory techniques for the measurement of vitamin D developed in the early 1980’s [73], investigators began to measure the vitamin D status of pregnant women. It became evident that women of darker pigment, who had migrated from sunnier climates to the United Kingdom or France, for example, and who wore clothing that left little skin exposed were found to be the most deficient [74,75,76,77,78,79]. Yet, it was thought for the most part that vitamin D deficiency was rare and could be avoided through some sunlight exposure and a daily vitamin D intake of 200 IU (5 μg) [80]. Therefore, it came as quite a surprise when the first report of widespread vitamin D deficiency in U.S. women of childbearing age was reported by the Center for Disease Control (CDC) in 2002 [81]. Using the Third National Health and Nutrition Examination Survey (NHANES III), 1988–94, it was found that 42% of African American women had serum 25(OH) concentrations below 15 ng/mL (37.5 nmol/L) [81]. Applying the current IOM definition of deficiency—25(OH)D level <20 ng/mL (50 nmol/L)—to the dataset increases the prevalence to ~75% [72]. 

Rates of deficiency reported during the past decade in the U.S. suggest that the degree of deficiency is greatest in those with darker pigmentation, *i.e.*, African American women, but deficiency exists among Hispanic and Caucasian women who have limited access to sunlight, either through limited activity outdoors, type of clothing, cultural practices, or thorough use of sunscreen when outdoors [70,82]. In two studies involving over 1000 pregnant woman in sunny South Carolina at 32°N, 75% African American, 33% Hispanic and 12% Caucasian women had frank deficiency [70,82], speaking to the extent and severity of this health problem. 

Another issue that plagues more industrialized nations is obesity. Women—including pregnant women, with a BMI greater than 30 are at increased risk of vitamin D deficiency [70]. The adipose tissue serves as a repository for vitamin D that does not get into the circulation. The problem may be further compounded by limited sunlight exposure and calorically rich but nutrient-poor diets such that multiple nutrients may be deficient, affecting both mother and her developing fetus.

In other areas of the world, deficiency also is commonplace, again reflecting a woman’s lifestyle, degree of skin pigmentation, where she lives (*i.e.*, the latitude and whether she is an urban dweller or lives in more rural areas), the time of year, and the most important factor—whether she has sunlight exposure [83]. Reports of profound deficiency among pregnant women, those with 25(OH)D concentrations <10 ng/mL (25 nmol/L) are common throughout the world: 18% of pregnant women studied in the UK [84], 25% in the UAE [85], 80% in Iran [86], 42% in northern India [87], 61% in New Zealand [88], 89.5% in Japan [89], and 60–84% of pregnant non-Western women in The Hague, Netherlands [90] had serum 25(OH)D concentrations <10 ng/mL (25 nmol/L). Interestingly, in a recent study involving 144 pregnant women in the greater Copenhagen area evaluated at 18, 32 and 39 weeks of gestation, 1.4–4.3% had this degree of deficiency [91]; this lower rate may correlate with increased dietary intakes of fish. For those areas of the world with higher rates of deficiency, it appears that a long-standing unawareness of how vitamin D is made and of the short and long-term health consequences of vitamin D insufficiency has led to widespread insufficiencies in most populations. 

## 9. Vitamin D During Pregnancy—Why Is It Important?

From the prior sections, it is clear that vitamin D deficiency during pregnancy is common throughout the world yet what effect does deficiency have on the mother and her developing fetus? There is a strong relationship between maternal and fetal (cord blood) circulating 25(OH)D levels [92,93,94,95] such that maternal vitamin D deficiency is mirrored by neonatal vitamin D deficiency. With severe maternal vitamin D deficiency, the fetus rarely may develop rickets *in utero* with manifestation at birth [96]. Such readily observable fetal and neonatal skeletal effects of profound vitamin D deficiency are easily understood in terms of cause and effect, but the more subtle effects of deficiency on the developing immune system, for example, and subsequent infection risk or immune dysfunction are more difficult to understand [19,84,97,98,99]. 

Vitamin D status during pregnancy appears to play a role in fetal skeletal development, tooth enamel formation, and general fetal growth and development [76,77]. Mannion *et al.* [100], comparing growth parameters in newborn infants with the maternal intakes of milk and vitamin D during pregnancy, found an association between vitamin D intake during pregnancy and birth weight. They reported with every additional 40 IU (1 μg) of maternal vitamin D intake, there was an associated 11-g increase in birth weight. Pawley and Bishop [101] in their study of 108 pregnant women and their offspring found a significant association between umbilical cord 25(OH)D concentrations and head circumference at 3 and 6 months’ postnatal age that persisted after adjusting for confounding factors. Maghbooli *et al.* found significantly wider posterior fontanelle diameter in neonates of mothers with vitamin D deficiency (as defined by a 25(OH)D level <34.9 nmol/L or ~14 ng/mL) compared to neonates whose mothers were not deficient [102]. Beyond growth, recent reports of neonates followed prospectively for acute viral infections and bronchiolitis from respiratory syncytial virus (RSV) support the premise that these states of deficiency do impact on the health of the young infant and suggest a greater role of vitamin D beyond bone health [103,104,105].

McGrath and others continue to investigate whether there are lasting effects of fetal and early infancy vitamin D deficiency on later adult disease processes such as anatomical changes of the brain, schizophrenia, multiple sclerosis, certain cancers, cardiovascular disease, and various other autoimmune diseases such as diabetes and lupus [106,107,108,109,110,111,112,113,114,115,116,117,118,119,120,121]. Because vitamin D status has not been a consistent concern during pregnancy, long-term data are sparse. The few studies that have been conducted have focused more on discernible neonatal effects of vitamin D during pregnancy, rather than the long-latency and later health effects. Reports of neonatal seizures due to severe hypocalcemia or rickets *in utero* that is manifested at birth from severe maternal and thus fetal vitamin D deficiency are rare and do not further our understanding of potential epigenetic effects of vitamin D [122,123]. During pregnancy, supplementation with the current standard amount of vitamin D in prenatal vitamins—400 IU (10 μg) vitamin D/day—has minimal effect on circulating 25(OH)D concentrations in the mother and her infant at term [6,124]. It is also known that infants of women who were deficient throughout pregnancy will reach a state of deficiency more quickly and with greater severity than infants of women replete during pregnancy [96]. 

While there are numerous epidemiological studies that bear evidence of the association between vitamin D deficiency and altered health, definitive proof in terms of randomized controlled trials is often lacking. For example, higher circulating 25(OH)D levels have been linked with improved glucose handling and beta-cell function [125], and a reduction in risk for a growing list of long latency diseases that include cardiovascular disease [57,126,127,128,129,130], multiple sclerosis [27,32,34], rheumatoid arthritis [36], systemic lupus erythematosus [25], type 1 and 2 diabetes [36], and various cancers [46,52,55,131,132,133,134,135,136], but critics counter that while such findings are intriguing, they do not provide definitive evidence of causality or a mechanism of action that come from randomized controlled trials, and may lead to what is referred to as “circular epidemiology” [137]. While we await the results of numerous clinical trials now underway to determine if there is a discernible effect of vitamin D in altering risk for various disease states to understand the role of vitamin D in health, it is important not to discount the mounting evidence from laboratory studies and prospective observation trials [103,104,105] that vitamin D—as a preprohormone—is essential in maintaining the immune system with profound implications [19,138,139,140]. 

## 10. Attaining Vitamin D Sufficiency: Sunlight and Dietary Supplementation

A recent study by Luxwolda *et al.* gives invaluable insight into vitamin D status of darker pigmented individuals living in a sun-rich environment in eastern Africa [141]. The investigators measured total (25(OH)D) concentrations of thirty-five pastoral Maasai (age 34 ± 10 years, 43% male); and twenty-five Hadzabe hunter-gatherers (age 35 ± 12 years, 84% male) living in Tanzania. Those participating in the study had skin type VI (darkly pigmented), wore a moderate degree of clothing, spent the major part of the day outdoors, and avoided direct exposure to sunlight when possible. The mean serum 25(OH)D concentration of the Maasai was 47.6 ng/mL (range 23.2–66.8 ng/mL or 119 nmo/L (range 58–167)). Similarly, the Hadzabe had a mean 25(OH)D concentration of 43.6 ng/mL (range 28.4–68.4) or 109 nmol/L (range 71–171). These concentrations were not related to age, sex or BMI. The 25(OH)D concentrations of the Maasai and the Hadzabe are on average more than double the concentrations of Africans living in the United States and other industrial countries.

With regard to safety, sunlight is superior to oral supplementation. One does not become vitamin D toxic from sunlight exposure; however, in comparison, people have become toxic from ingesting too much oral vitamin D. In an adult, it appears that the upper limit of tolerability of vitamin D is a daily consumption of thousands of international units of vitamin D—above 10,000 IU/day [142,143]. There is a safety mechanism in place with sunlight: sunlight-derived vitamin D triggers downregulation of certain enzyme systems and upregulation of others in the body to dispose of any vitamin D and its metabolites not needed by the body. Judicious sunlight exposure is not a clear cut entity; however, as too much sun exposure can lead to sunburn, photoaging, and skin cancer [144,145]. In addition, what amount of sunlight is sufficient to achieve optimal vitamin D status varies depending on a host of factors such as the time of day, the time of year, the latitude, degree of skin pigmentation, type and extend of clothing, body surface area exposed and one’s body mass index (BMI) [146]. Of note, in recent years, the health authorities in western countries have—to an increasing extent—warned against systematic sunlight exposure and solarium due to the well-documented side-effects and advocated regular use of sunscreen at sun exposure. A large proportion of the younger adult population may follow these guidelines, which may increase the risk of vitamin D deficiency. Since the modern diet supplies less than 10% of one’s total vitamin D requirements, if judicious sunlight exposure is not an option, then the only alternative for the pregnant woman is vitamin D supplementation.

## 11. Effectiveness of Vitamin D Supplementation During Pregnancy

There has been a paucity of studies evaluating the requirements and effects of vitamin D supplementation during pregnancy. The studies that were available in a Cochrane review more than a decade ago indicated that the Adequate Intake (AI) for vitamin D during pregnancy of 400 IU/day is grossly inadequate, especially with ethnic minorities [147]. As predicted by vitamin D pharmacokinetics, supplements of 1000 IU/day of vitamin D to pregnant women result in a 12.5 to 15.0 nmol/L increase in circulating 25(OH)D concentrations in both maternal and cord serum compared with nonsupplemented controls [75,76,77]. This premise is supported by our two randomized clinical trials involving vitamin D supplementation of pregnant women [6,148].

## 12. Results of Two Recent Randomized Controlled Trials During Pregnancy

**Figure 2:**
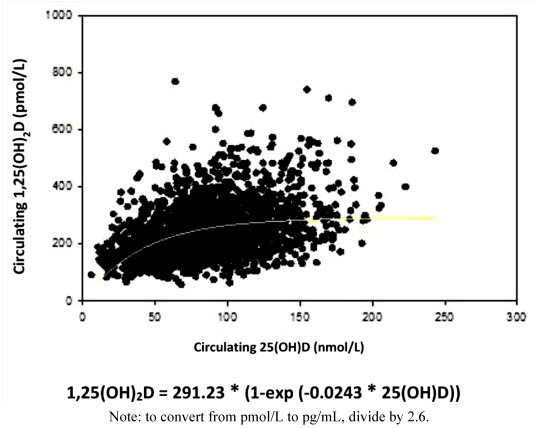
Relationship of circulating 25(OH)D on circulating 1,25(OH)D during pregnancy.

In our recently completed NICHD vitamin D supplementation trial involving a diverse group of pregnant women less than 16 weeks of gestation, 4000 IU (100 μg) vitamin D_3_/day was superior to 400 (control)- or 2000 IU/day in achieving circulating 25(OH)D of at least 40 ng/mL (100 nmol/L), the point at which 1,25(OH)_2_D begins to be optimized (see Figure 2) [6]. While 4000 IU/day was superior to 2000 IU/day in achieving the Institute of Medicine’s threshold for sufficiency of a 25(OH)D concentration of ≥20 ng/mL (50 nmol/L), it was not statistically significant—both 2000 and 4000 IU/day will achieve this level in pregnant women. If the goal, however, is to reach the point of 1,25(OH)_2_D optimization of 40 ng/mL (100 nmol/L), then there is a clear advantage of taking 4000 IU/day. While there was restriction of randomization in the NICHD trial necessary for initial IRB approval, such a restriction was not in place for the second pregnancy Thrasher Research Fund Trial (described below). Even in the latter trial where women were randomized to either 2000 IU (50 μg) or 4000 IU (100 μg) vitamin D_3_/day, there were no safety issues surrounding vitamin D supplementation and there was a trend where specific health complications of pregnancy such as preterm birth, preterm labor, hypertensive orders of pregnancy, gestational diabetes, and infection were higher in the 400 IU-compared with the 4000 IU-group, but did not reach statistical significance [149]. When analyzed together as comorbidities of pregnancy and controlling for race, there were statistically significant differences between the 400 IU-, the 2000 IU-, and the 4000 IU groups with fewer events in the 4000 IU group (*p* = 0.03) [149].

Consistent with the NICHD trial results, another recently completed study funded by the Thrasher Research Fund where women were randomized to either 2000 (50 μg)- or 4000 IU (100 μg) vitamin D_3_/day showed that the while the mean change from maternal 25(OH)D baseline was 12.2 ± 13.2 ng/mL in the 2000 IU group and 15.2 ± 12.9 ng/mL in the 4000 IU group (*p* = 0.16), the overall 25(OH)D rate of increase differed significantly between the two dose groups (*p* = 0.036) with a higher rate in the 4000 IU group. Mean infant 25(OH)D level (ng/mL) was 22.1 ± 10.3 in the 2000 IU group and 27.0 ± 13.3 in the 4000 IU group (*p* = 0.024). Secondary analysis indicated that preterm birth and labor were inversely related to pre-delivery vitamin D status (preterm birth *OR* = 0.50 per 10 ng/mL, *p* = 0.002; preterm labor or birth *OR* = 0.72 per 10 ng/mL, *p* = 0.012), an effect that persisted even after controlling for race. There were no adverse events associated with vitamin D supplementation [148]. 

Preliminary analysis of the two concurrent vitamin D pregnancy NICHD (*n* = 350) and Thrasher Research Fund (*n* = 157) trials was undertaken to assess the health characteristics and outcomes of this larger, combined group using a common data dictionary [150]. As noted above, in the NICHD trial, women were randomized to 400 IU (10 μg), 2000 IU (50 μg) or 4000 IU (100 μg)/day, stratified by race. In the Thrasher trial, participants were randomized to 2000 (50 μg) or 4000 IU (100 μg)/day, also stratified by race. All participants received study drugs from the same manufacturing lot. Studies administered identical questionnaires to produce comparable sociodemographic/clinical characteristics. Outcome measures included: (1) maternal baseline and delivery 25(OH)D; (2) neonatal 25(OH)D; (3) comorbidities of pregnancy: gestational diabetes, hypertensive disorders of pregnancy, infection—any, bacterial vaginosis (BV), and preterm birth without preeclampsia. In the combined cohort, there were 110 in the control group, 197 in 2000 IU group, and 189 in the 4000 IU group. The treatment groups differed on the basis of parity (*p* = 0.022); insurance status (Medicaid status more common in the 2000 IU group *p* = 0.006); and education (higher education level in the 400 IU group, *p* = 0.012). No differences were noted between groups in baseline 25(OH)D (*p* = 0.24), but differences between groups were noted in vitamin D status within one month of delivery (*p* < 0.0001) and in cord blood (*p* = 0.0001), with improved status in the 4000-IU group overall. No differences were noted between groups in terms of independent or combined comorbidities of pregnancy. There was a trend where preterm birth without preeclampsia was lower with increasing 25(OH)D concentration (Odds Ratio (*OR*) 0.83 (Confidence Interval (CI) 0.68–1.01); *p* = 0.057). The risk of combined comorbidities of pregnancy adjusted for race/ethnicity and study was significantly lower with increasing 25(OH)D concentration (Odds Ratio per 10 ng/mL increase in 25(OH)D: 0.84; 95% CI 0.74–0.96; *p* = 0.0095) [150].

The debate about what constitutes frank deficiency, insufficiency, and sufficiency continues [72,151]. The answer varies depending on the question—is the outcome bone integrity or immune function? There could be a different cut point for each category. Most would agree—including the Institute of Medicine in their most recent statement—that levels below 20 ng/mL (100 nmol/L) represent deficiency in the nonpregnant individual. There is less consensus with respect to pregnancy: based on our recent randomized controlled trial with pregnant women, it is clear that optimization of 1,25(OH)_2_D does not occur until total circulating 25(OH)D levels have reached 40 ng/mL (100 nmol/L) [6]. 

The significance of these trials is that vitamin D status is improved with 4000 IU vitamin D_3_ taken daily to achieve: (1) optimization of 1,25(OH)_2_D production, and (2) improved cord blood 25(OH)D concentration. Having a higher 25(OH)D concentration was associated with improved health outcomes in both studies, but whether improved vitamin D status is a marker of some other parameter or a direct effect of vitamin D supplementation remains unclear at this time. Studies specifically designed and powered to answer the question of whether or not vitamin D supplementation leads to improved health outcomes—lower risk of preterm birth, preeclampsia, and infection remain to be done. We can conservatively say that while we do not understand completely the role of 1,25(OH)_2_D during pregnancy, achieving optimal production can be done safely with 4000 IU vitamin D_3_/day, which appears to be the amount of vitamin D that would be conservatively generated by the body with adequate sunlight exposure over the duration of gestation.

## 13. Unanswered Questions and Direction of Future Research Endeavors

There are many unanswered questions about vitamin D’s “true” role during pregnancy: At no other time during the lifecycle is 25(OH)D so closely linked with 1,25(OH)_2_D [6]. As mentioned earlier, during the pregnant state, 1,25(OH)_2_D reaches levels many-fold higher than during the nonpregnant state, levels that would be toxic due to hypercalcemia to the nonpregnant individual, but which are essential during pregnancy. Why is calcium metabolism uncoupled from 1,25(OH)_2_D during this time? 

A leading theory is that 1,25(OH)_2_D is important for maternal tolerance to the foreign antigens of the fetus whose DNA is only half that of the mother’s, and in certain cases such as conception involving a donor egg—completely foreign. It is interesting that epidemiological studies involving pregnant women with preeclampsia—a clinical picture of inflammation and vasculitis—vitamin D deficiency has been implicated [152,153,154]. More work is warranted in this area to understand the possible role of vitamin D deficiency in preeclampsia.

The health effects data from vitamin D supplementation trials thus far, while tantalizing, are not conclusive in showing definitive “proof” for vitamin D as a potential candidate in the reduction of comorbidities of pregnancy, yet one cannot dismiss the strong correlation between reduced risk with increased serum 25(OH)D concentrations. Is vitamin D merely a marker of synergistic processes within the body? Is it a constellation of factors such as vitamin D acting in concert with vitamin A to create a healthful milieu? What about the interplay between genetics, epigenetics and daily fluxes in vitamin D status? Are those at greatest risk for vitamin D deficiency somehow protected by selective differences in vitamin D binding protein affinities and in the vitamin D receptor itself? If so, does substrate sufficiency—either through adequate sunlight exposure or vitamin D supplementation—saturate such effects, making them clinically irrelevant? What about vitamin D’s role in maintaining immune homeostasis during pregnancy? Looking carefully at the increasing number of epidemiological studies involving women with preeclampsia: is there a link with vitamin D deficiency where perhaps in certain women, there is loss of the vitamin D-mediated suppression of T cells that leads to a profound inflammatory reaction reminiscent of graft-*vs.*-host disease? In those women with a specific genetic repertoire—affinity for vitamin D and receptor processing—vitamin D deficiency may very well be the lost key to stopping the cascade of events. Only meticulous and well-designed clinical and translational science will lead to answers to these important questions. 

## 14. Conclusions

The role of vitamin D during pregnancy and its effect on maternal and fetal health is just beginning to be understood. In the last five years, there has been an explosion of published data concerning the immune effects of vitamin D, yet little is known in this regard about the specific immune effects of vitamin D during pregnancy. What is clear, however, is that vitamin D deficiency during pregnancy is rampant throughout the world, including countries such as the United States and Great Britain. While there remains much to be discovered and learned about vitamin D’s effect on the mother and her developing fetus, there is enough evidence to support the premise that deficiency is not healthful for either the mother or fetus. In this regard, the Institute of Medicine raised the 25(OH)D concentration from 10 ng/mL (25 nmol/L) to 20 ng/mL (50 nmol/L). A recent randomized controlled trial with 350 women of diverse racial and ethnic backgrounds showed that 4000 IU vitamin D/day is most effective in improving the vitamin D status of pregnant women, attaining circulating levels of at least 40 ng/mL (100 nmol/L) for 25(OH)D, and was necessary to achieve optimal 1,25(OH)_2_D production. The average level of circulating 25(OH)D achieved in 4000 IU group in this RCT was 50 ng/mL, and thus corresponds to what an average circulating 25(OH)D level has been shown to be in tribal Africans living in their native environment [141]. The novel finding from this trial that 25(OH)D concentration drives 1,25(OH)_2_D production during pregnancy sets the stage for additional research endeavors to delineate this relationship only found during pregnancy and to undercover its mechanisms of action and its putative role in maternal immune tolerance to the fetus. 
